# Revisiting promising preclinical intracerebral hemorrhage studies to highlight repurposable drugs for translation

**DOI:** 10.1177/1747493020972240

**Published:** 2020-11-13

**Authors:** Siobhan Crilly, Sarah E Withers, Stuart M Allan, Adrian R Parry-Jones, Paul R Kasher

**Affiliations:** 1 Division of Neuroscience and Experimental Psychology, Lydia Becker Institute of Immunology and Inflammation, School of Biological Sciences, Faculty of Biology, Medicine and Health, Manchester Academic Health Science Centre, The University of Manchester, Manchester, UK; 2 Division of Cardiovascular Sciences, Lydia Becker Institute of Immunology and Inflammation, School of Medical Sciences, Faculty of Biology, Medicine and Health, Manchester Academic Health Science Centre, The University of Manchester, Manchester, UK; 3 Manchester Centre for Clinical Neurosciences, Salford Royal NHS Foundation Trust, Manchester Academic Health Science Centre, Salford, UK

**Keywords:** Intracerebral hemorrhage, cerebrovascular disease, drug trials, translation, inflammation, deferoxamine, statins, Anakinra

## Abstract

Intracerebral hemorrhage is a devastating global health burden with limited treatment options and is responsible for 49% of 6.5 million annual stroke-related deaths comparable to ischemic stroke. Despite the impact of intracerebral hemorrhage, there are currently no effective treatments and so weaknesses in the translational pipeline must be addressed. There have been many preclinical studies in intracerebral hemorrhage models with positive outcomes for potential therapies *in vivo*, but beyond advancing the understanding of intracerebral hemorrhage pathology, there has been no translation toward successful clinical application. Multidisciplinary preclinical research, use of multiple models, and validation in human tissue are essential for effective translation. Repurposing of therapeutics for intracerebral hemorrhage may be the most promising strategy to help relieve the global health burden of intracerebral hemorrhage. Here, we have reviewed the existing literature to highlight repurposable drugs with successful outcomes in preclinical models of intracerebral hemorrhage that have realistic potential for development into the clinic for intracerebral hemorrhage.

## Introduction

Intracerebral hemorrhage (ICH) is the most severe subtype of stroke and is a leading global cause of mortality and adult disability. The extravasation of blood into the brain parenchyma results in the formation of a hematoma causing edema, tissue damage, and neuroinflammation responsible for neurological deficits and potentially fatal mass effect.^1^ The only current interventions for ICH patients continue to be limited to care on a stroke or critical care unit, reversal of anticoagulants (12%–20% of patients), blood pressure lowering to <140 mmHg, and surgical removal of the hematoma in carefully selected cases.^2,3^ Surgical intervention to aspirate the hematoma to a volume of <15 mL appears to show the best outcomes in patients with large hematomas^4^; however, the remaining blood and damaged tissue still needs to be treated.^1^ Patients with hematomas too small, deep, or disperse to surgically remove require an option of a medical treatment. In low-middle income countries that may not have specialized stroke care units, it is not always possible for hematoma evacuation, and different methods of surgical removal have shown mixed results in improving stroke outcomes.^4,5^ Continued efforts to identify effective medical treatments for ICH patients are therefore paramount.

To date, preclinical research in animal models of ICH has advanced understanding of the pathological mechanisms involved in brain injury. Some of this understanding has translated to clinical trials in ICH patients for the reduction of blood pressure,^6^ iron load,^7^ and neurovascular protection,^8^ without conclusive clinical benefit.^9^ Reduction of blood cholesterol using statins has been linked with increased ICH risk,^10^ but statins are also associated with improved outcomes after ICH.^11^ This has led to interest in statins as a treatment for ICH and for secondary prevention, and a phase III trial is planned to commence in 2020 (NCT03936361). Currently, interleukin-1 (IL-1) receptor antagonist (Anakinra) is in phase II trials for ICH^12^ based on previous successes in ischemic stroke and subarachnoid hemorrhage patients.^13,14^ Despite mounting evidence of medically targetable pathologies in ICH and the encouraging preclinical results for some treatments, as yet there has been no translation for a successful clinical therapy.

**Figure 1.**
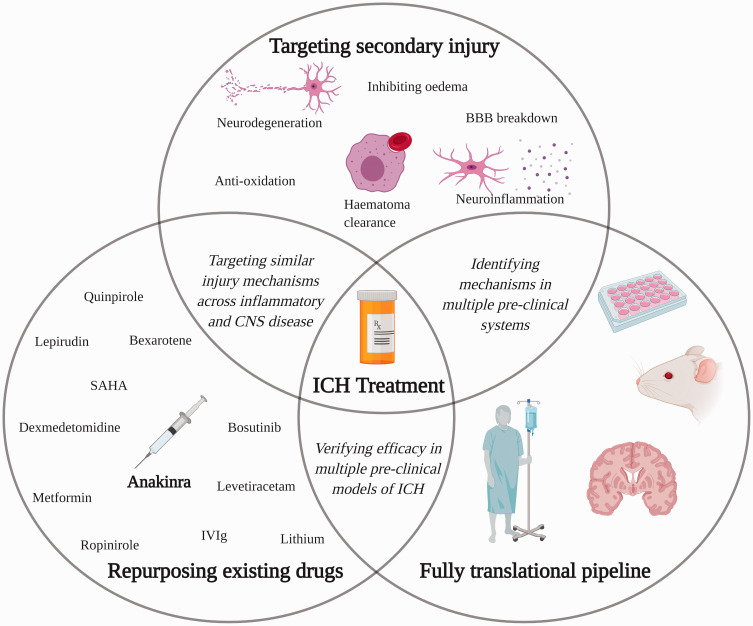
A diagrammatical representation of the necessary collaborative steps to translation of a medical therapeutic for ICH patients. ICH: intracerebral hemorrhage; BBB: blood–brain barrier; CNS: central nervous system; SAHA: suberoylanilide hydroxamic acid; IVIg: intravenous immunoglobulin.

Progressing drugs into the clinic requires multidisciplinary research, including the use of clinically relevant material such as patient blood, aspirated hematoma tissue, and postmortem brains for experimental target validation (Figure 1).^15^ Further emphasis on accurate translational preclinical strategies such as drug delivery methods and timing^16^ and confirmation in spontaneous ICH models is also required. There has been a historical trend for translational failure in stroke research,^17^ and as such there has been a general lack of enthusiasm in recent years from the pharmaceutical industry to support the development of novel compounds for stroke. The volume of ischemic stroke trials (1412) vastly outnumbers hemorrhagic stroke trials (142) but corresponds with very limited success. We need to approach translational research for ICH with a more realistic view and focus on clinically relevant preclinical investigations and the repurposing of approved drugs for ICH treatment.

Here, we review pharmacological interventions that show promise in preclinical *in vivo* studies that have repurposable potential (Table 1) that could be revisited for indications in clinical ICH treatment.

**Table 1.** A list of drugs shown to be beneficial in *in vivo* models of experimental ICH

					Improvements in parameters assessed								
Drug name	Mechanism of neuroprotection	Model	Timing of administration	Dosing	Behavior	Edema	Volume of hematoma	Brain atrophy	Neuro degeneration	Immune activation	Oxidative stress	*In vitro* study	Ref
Ambroxol	Anti-inflammation	Mouse/autologous blood	24 h post-ICH	35–70 mg/kg	yes	yes		yes	yes	yes			18
Ancrod	Hematoma clearance	Rat/collagenase	30 min post-ICH	10 or 30 IU/kg			yes						19
Bexarotene	Hematoma clearance	Mouse/autologous blood + collagenase	3 h post-ICH then once daily	5 mg/kg	yes		yes					yes	20
Bosutinib	Anti-inflammation	Mouse/ autologous blood	1 and 6 h post-ICH	1–25 mg/kg	yes	yes				yes			21
Cordycepin	Anti-inflammation	Mouse/autologous blood	30 min post-ICH	20 mg/kg	yes	yes		yes	yes	yes			22
Dexmedetomidine	Antioxidation	Mouse/autologous blood	1–3 days post-ICH	25 µg/kg	yes	yes	yes			yes			23
Dimethyl fumarate	Anti-inflammation	Mouse and rat/ autologous blood	2 h post-ICH then twice daily	15 mg/kg	yes	yes				yes			24
Edaravone	Anti-inflammation	Rat/collagenase	1 h post-ICH then daily	3 mg/kg	yes	yes		yes					25
EGb761	Inhibition of apoptosis	Mouse/collagenase	24 h post-ICH daily for 21 days	100 mg/kg	yes			yes				yes	26
Epicatechin	Antioxidation	Mouse/collagenase and autologous blood	3 h post-ICH and once daily for 3 days	15 and 45 mg/kg	yes	yes	yes	yes	yes		yes		27
Fimasartan	Anti-inflammation	Rat/collagenase	30 days pre-ICH	0.5–3 mg/kg	yes	yes	no	yes		yes		yes	28
Fisetin	Anti-inflammation	Mouse/collagenase	Daily for 3 days post-ICH	10–90 mg	yes	yes		yes		yes			29
Geranylgeranylacetone	Neuroprotection	Rat/collagenase	48 h pre-ICH	200–800 mg/kg	yes	yes	yes						30
Ghrelin	Anti-inflammation	Mouse/autologous blood	At time of ICH and 1 h post	10, 20 and 30 µg	yes	yes	yes	yes		yes			31
Ginkgolide B	Neuroprotection	Rat/autologous blood	30 m post-ICH and once daily up to 5 days	10–20 mg/kg				yes		yes			32
Glibenclamide	Anti-inflammation	Rat/autologous blood	Immediately post-ICH	10 µg/kg	yes	yes							33
Glutathione	Neuroprotection	Mouse/autologous blood	At ICH and daily for 3 days	50–200 mg/kg	yes	yes		yes					34
Glycine	Neuroprotection	Rat/autologous blood	1 h post-ICH	0.2–3 mg/kg	yes	yes	yes	yes					35
Glycyrrhizin	Neuroprotection	Rat/collagenase	20 m post-ICH	10–100 uM	yes	yes			yes			yes	36
Isoliquiritigenin	Anti-inflammation	Rat/collagenase	30 min post-ICH then 12, 24 and 48 h	10–40 mg/kg	yes	yes	yes		yes	yes			37
IVIg	Anti-inflammation	Mouse/collagenase	1 h post-ICH	0.5 and 2 g/kg	yes	yes	no			yes			38
Laropiprant	Anti-inflammation	Mouse/collagenase	1 h post-ICH	0.4 mg/kg	yes		yes						39
Lepirudin	Hematoma clearance	Rat/autologous blood	With blood injection	6 ATU		yes							40
Levetiracetam	Anti-inflammation	Rat/autologous blood	Not reported	50 mg/kg	yes	yes	yes	yes		yes		yes	41
Lithium	Inhibition of apoptosis	Rat/collagenase	3 days pre-ICH	2 mEq/kg	yes		no	yes		yes			42
Metformin	Neuroprotection	Rat/autologous blood	30 min pre-ICH and daily after	100 mg/kg	yes			yes		yes	yes		43
Nicotinamide mononucleotide	Antioxidation	Mouse/collagenase	30 min post-ICH		yes	yes	no	yes		yes	yes		44
Nrf2 agonist RS9	Antioxidation	Mouse/autologous blood	At injection and daily for 2 days	0.2 mg/kg	yes	yes		yes			yes	yes	45
Pinocembrin	Anti-inflammation	Mouse/collagenase	2 h post-ICH and twice daily	5 mg/kg	yes	yes	yes			yes		yes	46
Progesterone	Anti-inflammation	Mouse/collagenase	1, 6, 24 and 48 h post-ICH	8 mg/kg	no	yes	yes	yes	yes	yes	yes		47
Quinpirole + Ropinirole	Anti-inflammation	Mouse/autologous blood + collagenase	1 h post-ICH and daily	1–5 mg/kg	yes	yes	yes			yes			48
Rapamycin	Anti-inflammation	Rat/collagenase	1 h post-ICH	50, 150 and 450 mg/kg	yes	yes	yes	yes		yes			49
Resveratrol	Neuroprotection	Mouse/collagenase	30 min post-ICH	10 mg/kg	yes	yes	yes	yes		yes			50
Silymarin	Anti-inflammation	Mouse/collagenase	30 min post-ICH	200 mg/kg				yes		yes	yes		51
Simvastatin	Anti-inflammation	Mouse/collagenase	At time of ICH and once daily	0.1 µg/µl	yes	yes		yes		yes			52
Suberoylanilide hydroxamic acid (SAHA)	Neuroprotection	Mouse/collagenase	1 h post-ICH	70 mg/kg	yes			yes	yes	yes			53
Tauroursodeoxycholic acid (TUDCA)	Inhibition of apoptosis	Rat/collagenase	1 h pre- and 1-3 h post-ICH	10–200 mg/kg	yes		yes	yes		yes			54
Tempol	Antioxidation	Rat/collagenase	At time of ICH and 12, 36 and 60 h post	100 mg/kg	yes	yes		yes			yes		55
Theaflavin	Anti-inflammation	Rat/collagenase	1 day pre-ICH	25–100 mg/kg	yes	yes		yes		yes	yes		56
Tin-mesoporphyrin	Antioxidation	Pig/autologous blood	With blood injection	87.5 µM		yes	yes					yes	57

IVIg: intravenous immunoglobulin; ICH: intracerebral hemorrhage.

“Yes” means that the drug showed advantageous outcomes in these injury parameters; “no” means no change, or deleterious outcomes.

To collate evidence for the potential translation and repurposing of medical treatments from successful *in vivo* studies to the clinic for ICH, we analyzed the existing literature. Articles were identified from an NCBI search using the search terms “intracerebral hemorrhage,” “in vivo,” and “intervention” from 2000 until 2020 and are presented in Table 1. Reports of use of genetic interventions, biologics, or any regenerative medicine approaches or models of subarachnoid hemorrhage were excluded. Clinical trial records and human pharmacology data were used to identify drugs with repurposable potential. All studies included in Table 1 in this review investigated drugs and compounds that are currently in clinical and nonclinical human use, presently in clinical trial, or endogenous compounds.

### Repurposable drugs for ICH

Perhaps the most promising and exciting possibilities for ICH therapy lies with repurposing drugs that have already been approved for clinical use. As mechanisms of secondary injury, the immune response, oxidative stress, edema, apoptotic damage, iron chelation, blood–brain barrier (BBB) protection, and hematoma clearance pathways all provide targets for therapeutic strategy. Drugs that have been validated as safe and effective for conditions with related pathology, with established administration routes for human use require fewer trials and inspire more confidence from both clinician and patient. Table 1 highlights those that may be repurposable based on preclinical evidence for efficacy in experimental ICH outlined below.

### Strategies for neuroprotection

ICH causes tissue damage through the release of toxic blood compounds into the brain parenchyma, loss of circulation, and increased intracranial pressure. Preventing tissue injury in the hematomal penumbra limits white matter damage and functional deficits and so is a target for protective therapies.^58^ The bile acid tauroursodeoxycholic acid (TUDCA) is safe for humans and has been trialled as a treatment for insulin resistance, biliary cirrhosis, and improving vascular function. Furthermore, TUDCA has been discussed as a therapy for traumatic brain injury due to neuroprotective effects in preclinical studies.^59,60^ Bile acids are amphipathic molecules that can cross the BBB and derive from cholesterol catabolism, pathways that are known to be dysregulated in ICH patients.^61–63^ Some evidence has also shown that TUDCA can inhibit neuroinflammation, block translation of nitric oxide synthase and migratory capacity of microglia, a strategy that has been protective in experimental ICH. TUDCA prevents apoptosis by inhibiting BAX transport to the mitochondria and thus release of cytochrome C.^64^ In ICH patients, immediate delivery after injury may be protective, as cholesterol catabolism is dysregulated, and it has been suggested that increased total bile acids can be associated with better clinical outcomes after ICH.^65^ Preventing apoptosis and decreasing neuronal injury remains an important therapeutic strategy for stroke sufferers,^66^ and experimentally, TUDCA has shown beneficial outcomes in a range of neurological and other nonliver diseases.^67^ In Rodrigues et al.,^54^ the authors show smaller hemorrhage lesions and less brain damage in rats with two doses of TUDCA before and 6 h after collagenase injection, compared to controls. Further preclinical investigation should address the clinical link between elevated levels of bile acids and smaller hematoma volumes and validate mechanisms of neuroprotection using postmortem tissue.

Lithium, a mood stabilizer prescribed for bipolar disorder, has shown beneficial outcomes in preclinical rat models of ICH through both pretreatment^42^ and concurrent strategies.^68^ Kang et al.^42^ showed that lithium administered intraperitoneally for three days prior to a collagenase injection resulted in improved sensorimotor outcomes 48 h after ICH, decreased brain swelling at 72 h, and less brain atrophy at six weeks. Following on from this study, Liu et al. measured glycogen synthase kinase 3β (GSK3) inhibition to demonstrate a mechanism by which lithium treatment inhibits microglial migration and COX2 expression. The authors found improved cognitive function and neuroprotection with intraperitoneal injection of lithium at 2 h after autologous blood injection in rats and attributed this to GSK3 inhibition and mediation of glutamate-mediated excitotoxicity and neuronal death.^68^ More recent studies also demonstrated neuroprotection with lithium by increasing hematoma clearance in rats,^69^ ameliorating BBB^70^ and brain tissue damage^71^, and reducing white matter injury associated with ICH in a mouse model.^72^ Lithium is already safely used for clinical application and therefore presents an attractive therapeutic candidate for ICH patients. Further studies into inhibition of GSK3 with recombinant osteopontin^73,74^ and EGb761^26^ have also shown beneficial outcomes in experimental ICH models with reduced neuronal cell death, increased angiogenesis, and neural stem cell proliferation.

Flavonoids such as fisetin^29^ and pinocembrin^46^ have been implicated in the clinical management of cardiovascular disease.^75^ Humans consume between 1 and 2 g of flavonoids daily,^76^ and both showed beneficial outcomes on functional deficits and secondary neuroinflammation following experimental ICH. Pleiotropic effects of rapamycin, an antifungal metabolite and specific mammalian target of rapamycin inhibitor, also include ameliorating neurological damage following experimental ICH^49^ and other neurological disease. Rapamycin currently has clinical applications in cancer and so with more preclinical evidence could be repurposed for ICH. Clinically, polyphenol resveratrol has been implicated in protection for cancer^77^ and heart disease^78^ and can cross the BBB. In both mice^50^ and rat^79^ models of ICH, resveratrol improved neurological outcomes, cell death, and edema. Given that these compounds are naturally occurring, they hold potential for rapid development into preventative and protective therapies for ICH patients.

Ginkgolides are a class of drug isolated from the *Ginkgo biloba* tree and ginkgolide B specifically has been used to treat cerebrovascular disease as a selective antagonist of glycine receptors and platelet activating factor.^80^ It has neuroprotective activity against nitric oxides, has anti-inflammatory properties,^81^ stimulates the upregulation of heme oxygenase-1 (HO-1),^82^ and causes inhibition of apoptotic protein expression.^83^ Ginkgolide B has also been explored as a therapy for cerebral ischemia, increasing BBB permeability and reducing edema^84^ *in vivo*. Following Hu et al.'s^32^ 2011 study in rats, ginkgolide B has been studied in a small number of hemorrhagic stroke patients to investigate impact on reoxygenation of the brain, resulting in decreased intracranial pressure and improved cerebral perfusion pressure.^85^ A standard extract of *Ginkgo biloba* leaves, EGb761 containing a mix of compounds including ginkgolide B was shown to decrease neuronal apoptosis and improve the functional outcomes in experimental ICH mice.^26^

### Targeting neuroinflammation

The NOD-like receptor pyrin domain-containing protein 3 (NLRP3) inflammasome initiates the activation of inflammatory molecules triggering cell death and proinflammatory cascades. NLRP3 inhibition has been in development to treat inflammatory diseases and shows promise in preclinical ICH investigation. A number of drugs in Table 1 target the NLRP3 sensing molecule to prevent inflammasome activation. Pretreatment of animals with fimasartan, an angiotensin II receptor inhibitor used clinically to treat hypertension and heart failure, reduced activation of NLRP3, and protected animals from edema and neurological deficit.^28^ Glibenclamide, commonly used for treatment of type 2 diabetes mellitus, has been shown to protect the BBB after ICH in rats^33^ and mice^86^ by inhibiting the NLRP3 inflammasome and IL-1β release. Treatment with edaravone, approved for amyotrophic lateral sclerosis (ALS) patients, at the time of collagenase injection showed improved ICH pathology and functional outcomes in rats,^25^ and further studies identified that neuroprotection was similar to MCC950 treatment, an NLRP3 specific inhibitor, improving neurological function and neurodegeneration in a rat autologous blood model.^87^ It is important to note that collagenase injection results in an exacerbated neuroinflammatory response when compared to autologous blood injection and so could confound anti-inflammatory results. Similar anti-inflammatory results were found for both silymarin,^51^ a natural compound used for liver disease in a mouse collagenase model and cordycepin,^22^ currently in clinical trials for leukemia therapy (NCT00003005), in a mouse autologous blood model. As NLRP3 remains a target for inhibiting IL-1β production in many inflammatory diseases from atherosclerosis to cancer, small molecule inhibitors are still in the pipeline.

High-mobility group box 1 protein (HMGB1) is a proinflammatory DNA-binding molecule secreted by activated macrophages, monocytes, and dendritic cells in inflammatory conditions including stroke. Elevated levels observed in ICH patients correlate with an increase of other proinflammatory markers such as IL-6 and tumor necrosis factor-α (TNF-α), 10 day National Institutes of Health stroke scale score, and 3-month modified Rankin Scale scores.^88^ Ohnishi et al.^36^ demonstrated glycyrrhizin as an inhibitor of HMGB1, improving cerebral edema and behavioral performance in rats after ICH, however through different mechanisms to antibody intervention, as glucocorticoid receptors or nitric acid production were not significantly affected. Glycyrrhizin, a constituent of *Glycyrrhiza glabra* or liquorice root, is used in traditional medicine for alleviating bronchitis, gastritis, and jaundice, with anti-inflammatory and antioxidant properties.^89^ Progesterone previously identified as neuroprotective after ischemic stroke *in vivo* also inhibited HMGB1 expression and proinflammatory cytokines like IL-1β in experimental ICH.^47^ It appears from these studies that targeting HMGB1 release and action in ICH patients has clinical relevance and is a potential avenue for therapy.

Dexmedetomidine is a pain medication similar in function to clonidine, an agonist of α2-adrenergic receptors noted for the lack of respiratory depression.^90^ It has been approved for clinical trials for ICH therapy with a minimal sedation pain relief strategy but has not progressed since 2017 (NCT03207100). There has also been a trial proposed to examine the effect of perioperative dexmedetomidine infusion in aneurysmal subarachnoid hemorrhage cases to reduce the need for vasodilatory agent nimodipine but had no effect on subsequent vasospasms and infarction.^91^ Dexmedetomidine has previously been shown to have neuroprotective roles in rat hippocampus and prevent memory deficits associated with ICH^92^ and to decrease reactive oxygen species (ROS) release.^93^ Song and Zhang^23^ investigated the effect of dexmedetomidine on NLRP3-mediated anti-inflammatory properties and inhibition of IL-1β to alleviate secondary injury in mice.

Intravenous immunoglobulin (IVIg) injection is an approved therapy for autoimmune conditions such as immune thrombocytopenic purpura, Guillian-Barré syndrome, and chronic inflammatory demyelinating polyneuropathy as a broad acting anti-inflammatory agent. In experimental ICH, IVIg treatment attenuated mast cell activation, implicated in ICH pathology to release IL-6,^94^ resulting in less brain edema and neurological deficit.^38^ Another drug that shows repurposable potential is levetiracetam, an antiepileptic drug that alleviates inflammation, and in experimental ICH inhibits IL-1β and TNF-α expression.^41^

### Inhibition of oxidative stress

Oxidative stress following ICH from blood compounds and dying cells plays a key role in neurodegeneration and cell death.^95^ Nuclear factor erythroid 2-related factor (Nrf2) is a basic leucine zipper protein which regulates cellular resistance to reactive oxidants and the expression of HO-1 which protects against hemoglobin-related injury. HO-1 responds to cellular damage and breaks down toxic heme within the hematoma to produce biliverdin, iron, and carbon monoxide. These products result in inflammation, apoptosis, cell proliferation, and angiogenesis and when dysregulated in disease states, such as ICH injury, are toxic to cells.^96^ Epicatechin, a flavonoid that modulates the Nrf2 pathway, shows a reduction in early brain pathology in mice following ICH,^27^ and theaflavin that modulates NF-κB signaling cascades of proinflammatory molecules^56^ are found in common food sources hailed for their antioxidant properties and neuroprotection in cerebrovascular damage. Additionally ghrelin,^31^ an endogenous hormone, has both antioxidant and anti-inflammatory functions by inhibiting the NLRP3 inflammasome, a promising mechanism to target secondary brain injury in ICH.

Sugiyama et al.^45^ demonstrated direct agonism of Nrf2 which resulted in reduced secondary brain injury *in vivo* and the pathogenic mechanisms *in vitro*. Their study was subsequent to the positive outcomes in experimental ICH demonstrated by Nrf2 agonists dimethyl fumarate,^24^ a multiple sclerosis therapy, and nicotinamide mononucleotide,^44^ an endogenous derivative of niacin. Tin-mesoporphyrin,^57^ used for the prevention of hyperbilirubinemia, showed attenuation of HO-1 expression and action, preventing iron-mediated ROS release and improving functional outcomes in experimental ICH. Wanyong et al.^55^ demonstrated that tempol (MBM-02), a scavenger of peroxynitrite-derived free radicals, is neuroprotective in experimental ICH supporting data of similar outcomes in models of traumatic brain and spinal cord injury.^97,98^ Tempol has been in clinical trials for radiation-induced alopecia and cancer. Despite promising preclinical outcomes in rodent models, to progress to clinic successfully, validation for efficacy in spontaneous models and human tissue is necessary.^16^

Quinpirole and ropinirole are dopamine receptor D2 (DRD2) agonists and are both used experimentally and for the clinical management of Parkinson's disease symptoms. Ropinirole has also been identified to have clinically therapeutic effects in ALS through a DRD2-independent mechanism and reducing ROS.^99,100^ Zhang et al.^48^ demonstrated that both drugs showed anti-inflammatory effects in both mouse models by reducing IL-1β, microglia activation, and migration. Bosutinib is a src tyrosine kinase inhibitor commonly used to treat chronic myeloid leukemia and therefore has clinical potential for repurposing. In a mouse model of ICH, bosutinib inhibits salt-inducible kinase-2 (SIK-2), attenuates inflammation, and is associated with better pathological outcomes.^21^ Bosutinib has been related to treatment-associated vascular adverse effects such as cerebral ischemia, myocardial infarction, and pulmonary hypertension although with a milder frequency than other tyrosine kinase inhibitors. Clinical ICH investigation should be enacted with caution as patients commonly have other underlying vascular pathologies.

### Lipid metabolism

Modulating cholesterol levels after ICH injury has also been a target for experimental therapy. Cholesterol is a major component of myelin and so production is essential for neurological repair. Cholesterol also plays a key role in regulating immune responses and cellular metabolism.^101^ Laropiprant is a prostaglandin D2 receptor antagonist and was administered with niacin to reduce low-density lipoprotein cholesterol levels in patients; however, it was removed from market due to inefficacy. Preclinically, in a mouse model of ICH, laropiprant treatment attenuated hematoma volume, iron deposition, and neurological deficits.^39^ Statins, used to prevent cardiovascular disease by inhibiting 3-hydroxy-3-methylglutaryl coenzyme A, have been an area of contention in stroke therapy with mixed reports of clinical benefits. Simvastatin is reported to protect against BBB breakdown and inflammation-mediated apoptosis in mice after ICH^52^; however, it is clear that clinical therapy with statins requires further investigation. Targeting each of these pathways has benefits in experimental ICH; however, in order to determine whether combination therapies targeting multiple pathways would increase clinical impact, a novel strategy for ICH, potential drug interactions need to be understood.

### Hematoma clearance

Hematoma clearance is a highly attractive method of reducing injury outcomes^4^ limiting hematoma expansion, which has previously been attempted in the clinic using pioglitazone^102^ and accelerating reparative cellular responses. Several surgical removal techniques have now been considered; however, these are not consistently associated with improved mortality or long-term functional benefits.^103^ Bexarotene is a retinoid and modulates microglial responses following ICH to increase phagocytosis of the hematoma. Traditionally used as an antineoplastic agent, it is FDA approved for clinical use in cutaneous T-cell lymphoma. Bexarotene increases Axl expression on myeloid cells, a phosphatidylserine receptor that promotes a reparative and phagocytic phenotype in infiltrating macrophages after ICH.^20^

Ancrod is a defibrinogenating agent derived from Malayan pit viper venom, a thrombin-like serine protease and approved as a therapeutic for peripheral vascular disease.^104^ It has been trialled for ischemic stroke therapy with inconsistent outcomes,^105–109^ attributed to the induced formation of fibrin and microvascular occlusion observed *in vitro*.^110^ Elger et al.^19^ found a 50% reduction in ICH volume in rats treated with a low dose of ancrod instead of aggravating the bleed. Treatment started after collagenase injection, and effects were observed 24 h after injury; however, results were inconclusive about impact on levels of cerebral edema.

Lepirudin is a recombinant form of anticoagulant hirudin with one amino acid difference from the endogenous human protein. Used to treat heparin-induced thrombocytopenia in humans, lepirudin was withdrawn from use in the US and EU in 2012 for reasons unrelated to safety. Lepirudin binds α-thrombin inhibiting the cleavage of fibrinogen and the protease-activated receptor recognition site.^111^ There is no increased risk of stroke associated with treatment for myocardial infarction in patients in contrast to heparin treatment.^112^ Sun et al.^40^ found that recombinant hirudin administered into the hematoma modulated the expression of aquaporin proteins 4 and 9 and relieved edema in a rat autologous blood model of ICH.

### Other drugs of interest

Furthermore, other drugs that do not currently appear to have neurological or cerebrovascular targets may hold promise for clinical ICH therapy. Glutathione, an adjuvant given in radio- and chemotherapy, is an endogenous antioxidant compound and administration to a mouse autologous blood model of ICH protected mitochondria from stress damage.^34^ Geranylgeranylacetone,^30^ an antiulcer drug, and metformin,^43^ prescribed for metabolic syndrome, both protected against neurological deficits in rat models of ICH. Suberoylanilide hydroxamic acid^53^ for treatment of cutaneous T-cell lymphoma and ambroxol,^18^ an antioxidant mucolytic agent, equally resulted in beneficial outcomes on functional behavior, brain atrophy, apoptosis, and neuroinflammation in mouse models of experimental ICH. Repurposing safe medications for neurological diseases with multiple pathologies such as those seen in ICH is the fastest way to clinical benefit, and these novel therapeutic avenues, usually identified by unbiased drug screens, should be validated by clinical trial.

These *in vivo* investigations have advanced the understanding of pathological mechanisms involved in ICH, and we propose that many have the potential to be effective in ICH therapy and should therefore be investigated further in the context of the human condition and clinical application.

## Discussion

The current pipeline for preclinical investigation using *in vivo* models is not working for translation to the clinic for ICH patients^15^. Previous experience with ischemic stroke research and the failure to translate from preclinical trials has resulted in a lack of confidence in the progression to the clinic for ICH. There is a surplus of preclinical drug studies for interventions with therapeutic potential that lack the direct evidence for proving translational relevance. The best way to overcome these limitations is to produce quality preclinical research, with accurate reporting in valid models of ICH and related comorbidities. Continuity between groups and ensuring that all preclinical animal studies are reported according to the Animal Research: Reporting of In Vivo Experiments (ARRIVE) guidelines (now updated^113^) will help build clinical confidence in the quality and integrity of this research and support the progression to translation. In cases where preclinical studies have not fully adhered to the ARRIVE guidelines (including appropriate reporting of use of male and female animals, randomization, sample sizes, blinding, etc.), further preclinical study may be necessary to validate efficacy before advancing to clinical application. Collaborations between preclinical scientists, pathologists, neurologists, and neurosurgeons are all essential to benefit the global clinical burden of ICH, and large rigorous preclinical studies without translation are wasteful and unethical. Therefore, back translation from patients, collection of postmortem samples, and a combination of multispecies *in vivo* and *in vitro* modeling need to be employed to promote a fluid pipeline of therapeutic investigation rather than a unidirectional approach for preclinical drug discovery.^16^ Investigation into drugs that are already approved for clinical use is less risky than newly developed compounds with undetermined activity. Repurposing safe drugs that are already clinically used likely represents the fastest and most effective way of addressing the need for medical ICH interventions.

Hemorrhagic stroke subtypes differ vastly in causation and pathology and are fundamentally opposite from the ischemic stroke condition. However, the blanket term “stroke” implies that they are more similar than in reality. Research into blood lipids suggests there are unique patient differences associated with risk.^114^ Many clinical and preclinical trials for ICH have previously been modeled in ischemic stroke conditions, with limited success. Some unique features of ICH pathology such as the hematoma cavity and the breach of the BBB perhaps offer exciting pathways of investigation, distinctive from ischemic stroke. Distancing ICH research from ischemic stroke could offer a different and beneficial perspective for future treatment strategies.^16^

Perhaps the most promising new candidates for future translation appear to be both inhibition of HMGB1 release and agonism of the Nrf2 antioxidant pathway. HMGB1 has been associated with both pathological proinflammatory roles and astrocyte-mediated angio- and neurogenesis in recovery. Inhibition of release and action is antioxidative and prevents edema in experimental ICH, and it can be targeted by naturally occurring therapeutics (glycyrrhizin and progesterone). It is clear that inhibition of the inflammatory response following ICH is beneficial to pathological outcomes; however, further clinical investigation is necessary to determine what the long-term effects of immune modulation are on recovery mechanisms, and how crucial timing of administration following ICH should be addressed. Many naturally occurring, endogenous, repurposable interventions have also been identified to upregulate the Nrf2 pathway of antioxidation and neuroprotection, alleviating pathogenic mechanisms following ICH. Dimethyl fumarate, therapy for inflammatory conditions such as multiple sclerosis and psoriasis, and one such Nrf2 inhibitor, has shown preclinical ICH benefit in extensive preclinical investigation. It could be that a combination therapy targeting anti-inflammatory, antiapoptotic, and antioxidant pathways may result in a sufficient reduction in brain injury in patients; however, this is still yet to be studied. Sometimes, promiscuity of drugs, such as those naturally occurring medicines with multiple active compounds, can be a virtue and target multiple pathological outcomes at once without complete understanding of the mechanism. Ultimately, a medical therapeutic to limit clinical secondary brain injury, paired with excellent clinical care, is the best strategy of treating all ICH patients and may have already been identified in the preclinical studies above. Collaborative strengthening of the translational pipeline is paramount for seeing these *in vivo* studies to completion and alleviating the global health burden of ICH.
